# *RNF115* deletion inhibits autophagosome maturation and growth of gastric cancer

**DOI:** 10.1038/s41419-020-03011-w

**Published:** 2020-09-26

**Authors:** Riyong Li, Zhaohui Gu, Xuan Zhang, Jiahong Yu, Jinqiu Feng, Yaxin Lou, Ping Lv, Yingyu Chen

**Affiliations:** 1 Department of Immunology, School of Basic Medical Sciences, Peking University; NHC Key Laboratory of Medical Immunology (Peking University), Beijing, China; 2 Center for Human Disease Genomics, Peking University, Beijing, China; 3 Medical and Healthy Analytical Center, Peking University, Beijing, China

**Keywords:** Macroautophagy, Gastric cancer

## Abstract

Autophagy is a highly conserved lysosome-dependent degradation system in eukaryotic cells. This process removes long-lived intracellular proteins, damaged organelles, and recycles biological material to maintain cellular homeostasis. Dysfunction of autophagy triggers a wide spectrum of human diseases, including cancer and neurodegenerative diseases. In the present study, we show that RNF115, an E3 ubiquitin ligase, regulates autophagosome–lysosome fusion and autophagic degradation under both nutrient-enriched and stress conditions. Depletion of the *RNF115* gene caused the accumulation of autophagosomes by impairing fusion with lysosomes, which results in an accumulation of autophagic substrates. Further investigation suggests that RNF115 interacts with STX17 and enhances its stability, which is essential for autophagosome maturation. Importantly, we provide in vitro and in vivo evidence that *RNF115* inactivation inhibits the tumorigenesis and metastasis of BGC823 gastric cancer cells. We additionally show that high expression levels of *RNF115* mRNA correlate with poor prognosis in gastric cancer patients. These findings indicate that RNF115 may play an evolutionarily conserved role in the autophagy pathway, and may act to maintain protein homeostasis under physiological conditions. These data demonstrate the need to further evaluate the potential therapeutic implications of RNF115 in gastric cancer.

## Introduction

Autophagy is a complex biological process that is crucial for maintaining cellular homeostasis^1^. Autophagy is a multistep process, initiated by autophagosome formation, followed by docking and fusion of the autophagosome with the endosome/lysosome to form the autolysosome, resulting in the elimination of engulfed substrates and eventual reformation of the autophagic lysosome^2^. The mechanisms of autophagy initiation and autophagosome formation have been clearly described, but the mechanisms of autophagosome–lysosome fusion remain to be elucidated.

In eukaryotic cells, protein degradation is primarily mediated through the ubiquitin proteasome system (UPS) and autophagy, and there is extensive cross talk and interplay between the UPS and autophagy^3^. In addition, many E3 ubiquitin ligases participate in regulation of autophagy, including NEDD4^4^, BRUCE^5^, Parkin^6^, and RNF185^7^. RNF115 (Rabring7 in mice) is a highly conserved protein, and is a RAB7 target protein. RNF115 directly binds the GTP-bound form of Rab7, and can be recruited efficiently to the late endosome/lysosome^8^. Moreover, RNF115 regulates the endosomal sorting of the EGFR protein, and knockdown of *RNF115* decreases the number of multivesicular bodies (MVBs)^9^. Functional MVBs are required for autophagic clearance of protein aggregates^10^. In addition, RNF115 participates in innate immunity and can target the HIV-I virus for lysosomal degradation^11–13^.

In the present study, we demonstrate that RNF115 regulates autophagy and promotes autophagosome–lysosome fusion by interacting with the STX17 protein. We also show that blocking autophagy by inactivating *RNF115* inhibits the growth of gastric cancer cells in vitro and in vivo, which maybe a potential therapeutic target for cancers.

## Results

### Knockdown of *RNF115* impairs autophagic flux

**Fig. 1:**
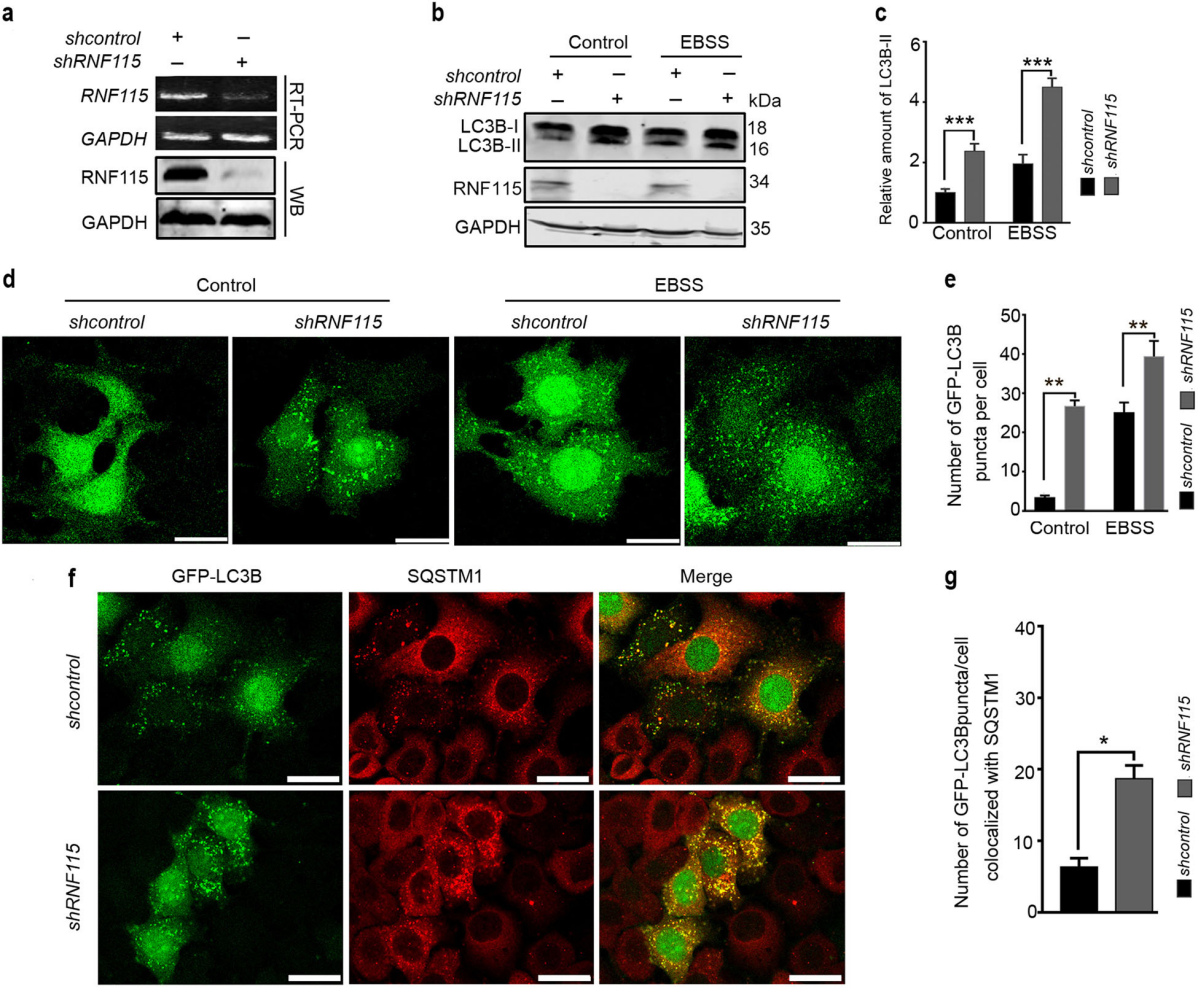
Depletion of RNF115 casued autophagosome accumulation. **a** RT-PCR and western blotting detected the levels of *RNF115* in Hela cells. **b**, **c** HeLa cells were transfected with *shcontrol* or *shRNF115* for 48 h, with or without EBSS for another 2 h, then LC3B-II levels were analyzed by western blotting. The relative amount of LC3B-II levels relative to GAPDH was analyzed. Average value in *shcontrol*-transfected cells without EBSS was normalized as 1. Data are means ± s.d. of results from at least three independent experiments. **d**, **e** Representative confocal microscopy images of GFP-LC3B distribution in stable GFP-LC3B Hela cells transfected with *shcontrol* or *shRNF115* for 48 h, and treated with or without EBSS for another 2 h. The number of GFP-LC3B puncta/cell was calculated. Data are means ± s.d. of at least 50 cells scored. **f**, **g** Representative confocal microscopy images were shown in stable GFP-LC3B HeLa cells transfected with *shcontrol* or *shRNF115* for 48 h, stained with anti-SQSTM1 antibody, and then observed by confocal microscopy. The number of GFP-LC3 puncta/cell colocalized with SQSTM1 aggregates was calculated. Data are means ± s.d. of at least 50 cells scored. Scale bar, 25 μm. *p < 0.05; **p < 0.01; ***p < 0.001.

To investigate the physiological effects of *RNF115* knockdown on the regulation of autophagy, experiments were conducted in *RNF115*-depleted cells. Using RT-PCR and western blot to evaluate the expression of gene and protein products, an effective shRNA against *RNF115* (*shRNF115*) was identified (Fig. 1a). Data from experiments proved that depletion of *RNF11*5 elevated the levels of endogenous LC3B-II protein (Fig. 1b, c) under both nutrient-rich and starvation conditions, compared with *shcontrol*-transfected cells. In the presence of rapamycin, the knockdown of *RNF115* also increased the accumulation of LC3B-II (Supplementary Fig. S1a). Similar results were observed in HEK293T, BGC823, and MCF7 cell lines (Supplementary Fig. S1b, d, f). In line with these results, *RNF115* knockdown increased the number of GFP-LC3B puncta per cell compared with the *shcontrol* group in HeLa cells. Autophagosome/autolysosome accumulation is also identified by colocalization of GFP-LC3B and P62/SQSTM1. Knockdown of *RNF115* led to a significant increase in the number of GFP-LC3 puncta colocalized with SQSTM1 per cell (Fig. 1f, g). These data show that *RNF115* knockdown increased the accumulation of autophagosome/autolysosome.

**Fig. 2:**
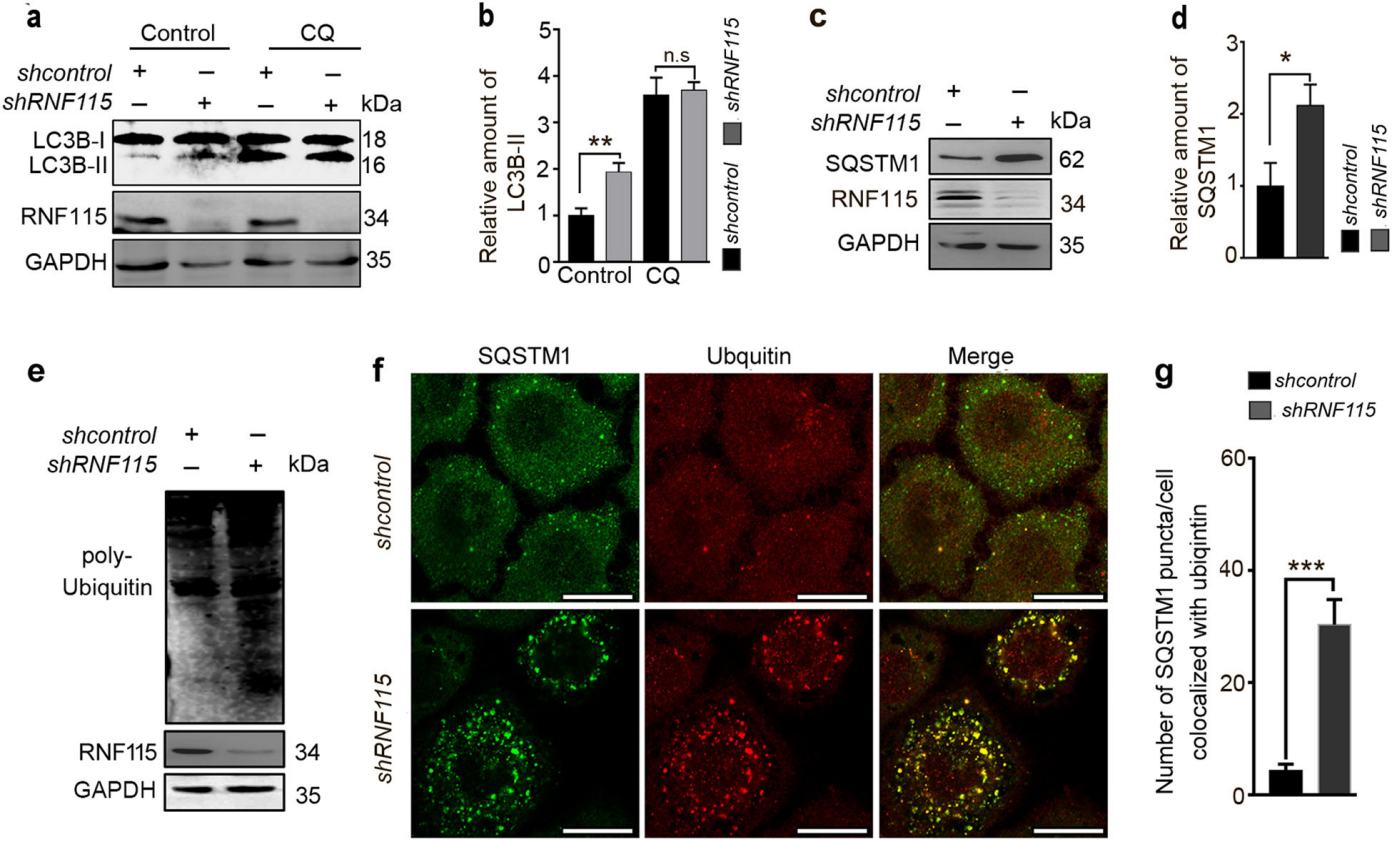
Depletion of RNF115 blocks autophagic flux. **a**, **b** HeLa cells were transfected with *shcontrol* or *shRNF115* for 48 h, with or without CQ (50 μM) for 4 h, then the levels of LC3B-II were analyzed by western blotting. The quantification of LC3B-II levels relative to GAPDH was analyzed. Average value in *shcontrol*-transfected cells without CQ was normalized as 1. Data are means ± s.d. of results from at least three independent experiments. **c**, **d** HeLa cells were transfected with *shcontrol* or *shRNF115* for 48 h, the SQSTM1 levels were analyzed by western blotting. The quantification of SQSTM1 levels relative to GAPDH was analyzed. Average value in *shcontrol*-transfected cells was normalized as 1. Data are means ± s.d. of results from at least three independent experiments. **e** Cells were treated as in **c**, the levels of ubiquitinated protein were detected by western blotting. **f**, **g** Hela cells were transfected with *shcontrol* or *shRNF115* for 48 h, stained with anti-SQSTM1 and anti-ubiquitin antibodies, and observed by confocal microscope. The number of SQSTM1 puncta/cell colocalized with ubiquitin aggregates was calculated. Data are means ± s.d. of at least 50 cells scored. Scale bar, 25 μm. *p < 0.05; **p < 0.01; ***p < 0.001; n.s not significance.

The increased levels of LC3B-II could be caused by either elevated autophagosome formation or blockade of autophagic flux^14^. To distinguish between these two possibilities, *RNF115* knockdown cells were treated with chloroquine (CQ), which blocks autophagosome–lysosome fusion, thereby causing an accumulation of autophagosomes^15^. As shown in Fig. 2a, b, the levels of LC3B-II were not further increased in *RNF115*-depleted cells treated with CQ compared with control cells, indicating that the loss of *RNF115* may block autophagic flux. We further examined the levels of autophagic substrates in *RNF115* knockdown cells. SQSTM1 is a well-characterized autophagic substrate that mediates the formation and autophagic degradation of ubiquitin-positive protein aggregates^16^. In *RNF115*-depleted HeLa cells, levels of SQSTM1 and ubiquitinated proteins were increased (Fig. 2c–e). The accumulation of SQSTM1 was also presented in *RNF115*-silenced HEK293T, BGC823, and MCF7 cell lines (Supplementary Fig. S1c–e). Confocal microscopy revealed that SQSTM1 and ubiquitin-positive aggregates were increased and colocalized (Fig. 2f, g). In addition, in RNF115-overexpressing HeLa cells, the levels of endogenous LC3B-II protein and SQSTM1 protein were reduced (Supplementary Fig. S2a), indicating that RNF115 may promote the rate of autophagic flux and/or increase the degradation of autophagic substrates. At the same time, we performed a recovery experiment in *RNF115*-depleted HeLa cells. Accumulation of SQSTM1 protein was significantly decreased after transfection with the RNF115 expression plasmid in *RNF115*-silencing cells (Supplementary Fig. S2b), implying that *RNF115* ablation modulated autophagy directly. Collectively, these findings suggest that the loss of *RNF115* impairs autophagic flux in mammalian cells.

### *RNF115* inactivation impairs autophagosome–lysosome fusion

**Fig. 3:**
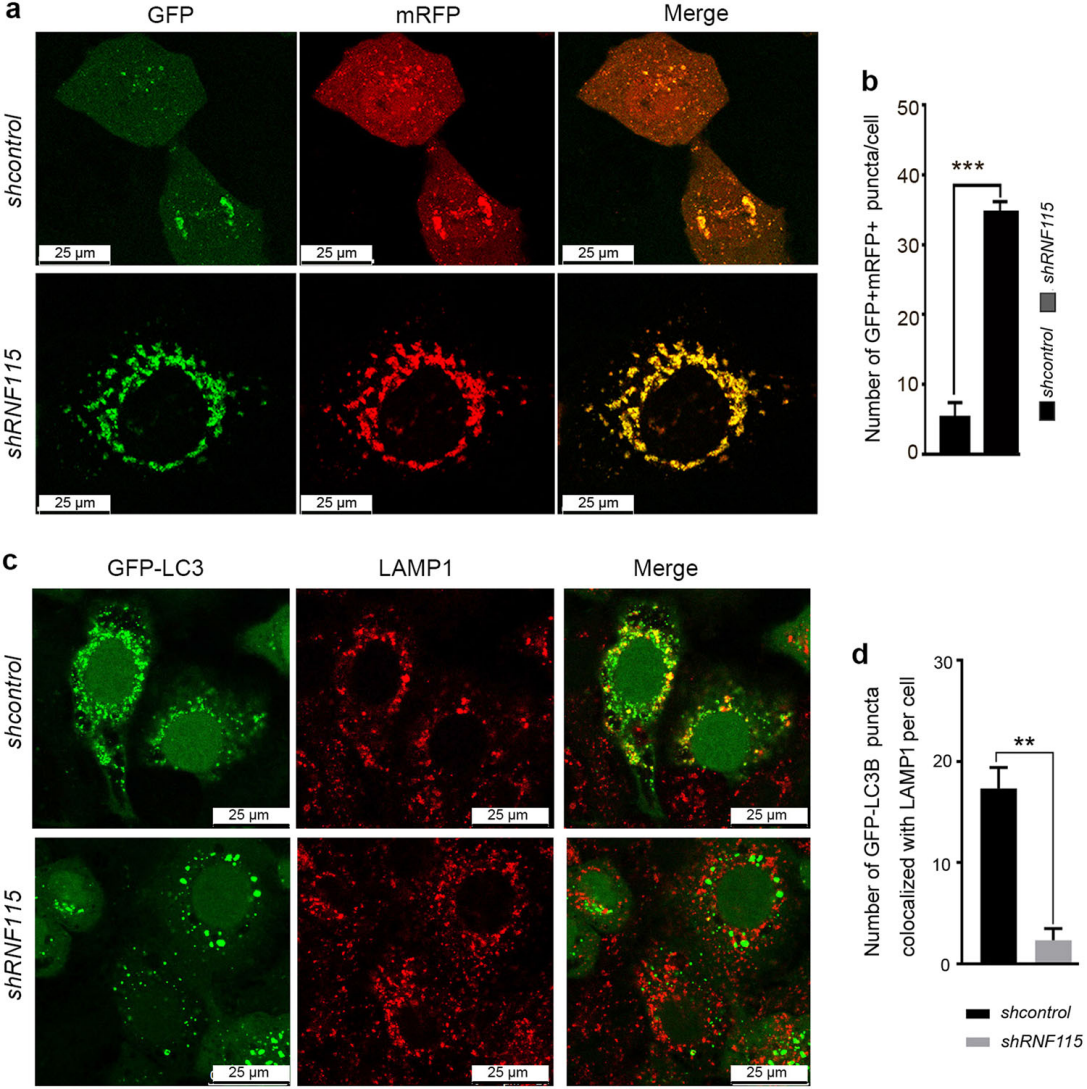
Knockdown of RNF115 impairs the fusion of autophagosome with lysosome. **a**, **b** HeLa cells were transfected with *shRNA* or *shRNF115* for 24 h, then with mTagRFP-GFP-LC3 for another 24 h and EBSS treatment for 2 h before confocal microscopy analysis. The quantification of GFP^+^RFP^+^ puncta/cell was calculated. Data are means ± s.d. of at least 50 cells scored. **c**, **d** GFP-LC3 stably expressing HeLa cells were cultured on confocal dishes and transfected with *shcontrol* or *shRNF115* for 48 h and EBSS treatment for 2 h. Then cells were stained with anti-LAMP1, and observed by confocal microscopy. The number of GFP-LC3 puncta/cell colocalized with LAMP1 was calculated. Data are means ± s.d. of at least 50 cells scored. Scale bar, 25 μm. **p < 0.01; ***p < 0.001.

We next determined at which step in autophagic flux is impaired by *RNF115* knockdown. ZFYVE1/DFCP1-labeled omegasomes, which act as cradles for autophagosome formation, exhibited no difference in control vs. *RNF115* knockdown cells (Supplementary Fig. S3a, b). Next, the expression of the tandem fluorescent-tagged LC3 (mTagRFP-GFP-LC3) was examined to monitor autophagosome–lysosome fusion (autophagosome maturation). In this assay, the fluorescent signal of GFP is quenched in the lysosome, while RFP shows more stable fluorescence in acidic compartments^17^. Therefore, autophagosomes and amphisomes are labeled with yellow, while red fluorescence signals are detected in autolysosome. As shown in Fig. 3a, b, we observed more yellow (RFP^+^GFP^+^) puncta in *RNF115*-depleted HeLa cells after EBSS treatment, compared with control cells. Simultaneously, *RNF115* deletion significantly decreased colocalization of GFP-LC3 with LAMP1 (Fig. 3c, d). These data suggested that *RNF115* knockdown suppressed the autolysosome–lysosome fusion in the autophagy process.

### RNF115 interacts with STX17 and enhances its stability

**Fig. 4:**
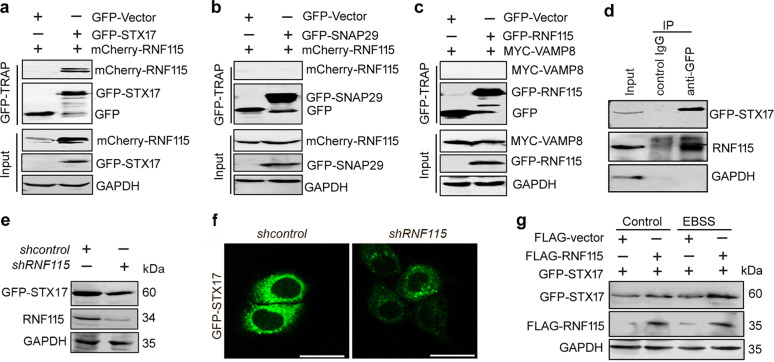
RNF115 interacts with STX17 and enhances its stability. **a**–**c** HEK293T cells were cotransfected with indicated plasmids for 24 h, then the cell lysates were subjected to GFP-TRAP beads. The interactions were analyzed by western blotting with anti-RNF115, anti-GFP, or anti-MYC antibodies, respevtively. GAPDH was used as the loading control. **d** HEK293T cells were transfected with GFP-STX17 for 24 h, the cell lysates were subjected to IP using either an anti-GFP antibody or an IgG isotype control. The interaction was analyzed by western blotting with anti-RNF115 antibodies. GAPDH was used as the loading control. **e** Hela cells were cotransfected with GFP-STX17 and *shcontrol* or *shRNF115* for 24 h, the levels of GFP-STX17 were analyzed by western blotting. **f** Cells were treated as in **e**, the fluorescence of GFP-STX17 was analyzed by confocal microscope. Scale bar, 25 μm. **g** HEK293T cells were cotransfected with indicated plasmids for 24 h, with or without EBSS for another 2 h, then the levels of GFP-STX17 were analyzed by western blotting.

The fusion of autophagosomes with lysosomes requires the concerted actions of SNARE proteins, Rab GTPases, and tethering factors^18^. RNF115 binds the GTP-bound form of RAB7 at the N-terminal portion, and acts as a RAB7 effector^8^. Thus, we explored whether RNF115-regulated autophagosome maturation involved SNARE complexes. Using GFP-TRAP assays, we found that GFP-STX17 precipitated mcherry-RNF115 and endogenous RNF115 (Fig. 4a, d). However, RNF115 failed to interact with SNAP29 and VAMP8 protein (Fig. 4b, c). Further evaluation indicated that depletion of *RNF115* downregulated the expression of STX17 protein (Fig. 4e, f). By contrast, RNF115 overexpression increased the accumulation of STX17 protein under both nutrient-rich and starvation conditions (Fig. 4g). We next measured the half-life of STX17 using the protein translation inhibitor cycloheximide (50 μg/ml) in *RNF115*-silenced cells. As shown in Supplementary Fig. S4, knockdown of *RNF115* promoted the decay of STX17 protein comparing with that control group. These data suggest that RNF115 might regulate the homeostasis of the STX17 protein.

Protein ubiquitination is associated with the destabilization and degradation of proteins. Therefore, we examined whether STX17 instability caused by *RNF115* knockdown was associated with ubiquitination degradation. Immunoprecipitation and western blot analysis showed that RNF115 did not significantly affect either ubk48-linked or ubk63-linked STX17 polyubiquitination levels (Supplementary Fig. S5), implying that another mechanism may be involved in the downregulation of STX17 in the *RNF115*-silenced cells.

### *RNF115* deletion inhibits the tumorigenesis and metastasis of BGC823 cells

RNF115 is highly expressed in ERα-positive breast cancer cell lines and tumors, and positively regulates the growth of breast cancer cells^19,20^. However, the function of RNF115 in other cancers is unknown. The Gene Expression profiling Interactive Analysis database^21^ showed that the expression levels of *RNF115* mRNA in patients with gastric carcinoma is higher than in the normal population (Supplementary Fig. S6a). Using the Kaplan–Meier Plotter online database (http://kmplot.com/analysis/index.php?p=service&cancer=gastric), we analyzed the correlation between *RNF115* mRNA levels and survival time in 876 patients, with gastric cancer. Low levels of *RNF115* mRNA correlated with better overall survival in gastric cancer patients (Supplementary Fig. S6b), indicating that decreased expression of RNF115 may be a favorable prognostic indicator in patients with gastric cancer. The expression trend of *STX17* is similar to that of *RNF115* (Supplementary Fig. S6c, d). Correlation analysis (http://gepia.cancer-pku.cn/detail.php?gene=&clicktag=boxplot) suggested a positive correlation between RNF115 and STX17 expression in gastric cancer (Supplementary Fig. S6e). Data from tissue microarray and immunohistochemistry demonstrate that RNF115 protein displayed moderate or high expression levels in most gastric adenocarcinoma cells (Supplementary Fig. S6f). RNF115 protein was mainly located in the cell cytoplasm of the mucosa gland, and exhibited a diffuse expression pattern.

**Fig. 5:**
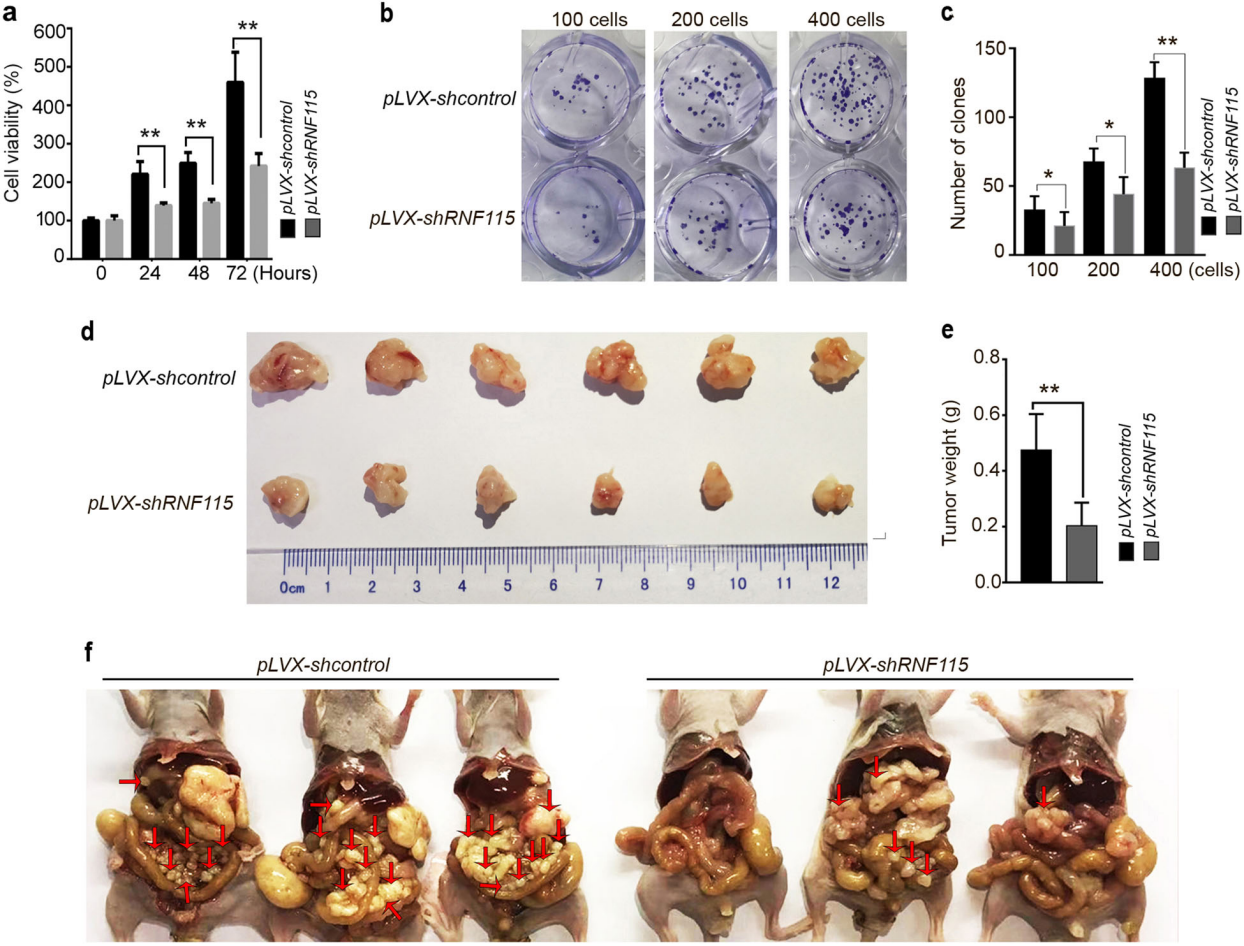
RNF115 knockdown in BGC823 cells inhibits cell growth, reduces tumorigenicity and metastasis. **a** BGC823 cells stable infected with *pLVX-shcontrol* or *pLVX-shRNF115* were seeded in 96-well plates (3 × 10^3^ cells/well; five replicates), serum starved for 18 h and then pulsed with 10% FBS for indicated time. Cell viability was detected. Data are means ± s.d. of results from three independent experiments. **b** Representative images of colony formation by indicated cells. **c** Number of clones counted in three independent experiments. **d**, **e** BGC823 cells stable infected with *pLVX-shcontrol* or *pLVX-shRNF115* (4 × 10^6^ cells/mouse) were injected subcutaneously in BALB/c nude mice. At day 19, excised xenograft tumors were imaged and weighed (*n* = 6). **f** BGC823 cells stable infected with *pLVX-shcontrol* or *pLVX-shRNF115* (6 × 10^6^ cells/mouse) were injected into the abdominal cavity of mouse. At day 28, the mice were sacrificed and the metastatic nodules was observed and photographed. Arrows indicate the disseminated tumor nodules. *p < 0.05; **p < 0.01.

To further explore the role of RNF115 in the development and progression of gastric cancer, we constructed lentiviral knockdown vector, *pLVX*-*shRNF115*, and screened BGC823 cell lines for stable knockdown of *RNF115*. qRT-PCR and western blot analysis confirmed that the *RNF115* mRNA and protein were significantly decreased in BGC823 cells, respectively (Supplementary Fig. S7a, b). In addition, the accumulation of both SQSTM1 and LC3B-II was observed by western blot analysis, and CQ treatment failed to affect the levels of these proteins (Supplementary Fig. S7c), indicating the blockade of autophagic flux in *RNF115*-depleted BGC823 cells. Furthermore, cell viability and colony formation assays suggested that knockdown of *RNF115* arrests the growth of BGC823 cells (Fig. 5a–c).

The in vivo effects of RNF115 were evaluated using a gastric cancer xenograft model established in BALB/C nude mice. These mice were subcutaneously injected with *pLVX*-*shcontrol*/BGC823 cells or *pLVX*-*shRNF115/*BGC823 cells. The control group developed grossly visible tumors at the site of injection (Fig. 5d). By comparison, the *pLVX-shRNF115* group displayed smaller tumors. The tumor weight in the *pLVX-shRNF115* group was significantly lower than that in the control group (Fig. 5e), suggesting that knockdown of *RNF115* inhibits the growth of BGC823 cells in vivo.

We further established an animal model of peritoneal metastasis by injection of *pLVX*-*shcontrol*/BGC823 cells or *pLVX*-*shRNF115/*BGC823 cells into mice to explore the effects of RNF115 on tumor metastasis. All mice were euthanized 28 days after injection. A large number of metastatic nodules were found in the peritoneal cavity in control mice (Fig. 5f). By contrast, the *pLVX*-*shRNF115* group had few or no tumor nodules. These data suggested that the inactivation of *RNF115* might prevent peritoneal homing of gastric cancer cells and growth of metastatic nodules.

## Discussion

In this study, we demonstrate that RNF115 is a positive regulator that participates in the autophagosome–lysosome fusion. Inactivation of *RNF115* impaired autophagy, and caused autophagic substrate accumulation under basal or starvation conditions. We report that RNF115 interacts with STX17 and enhances its stability, which can promote autophagosome maturation. In addition, *RNF115* silencing can inhibit the tumorigenicity and metastasis of gastric cancer cells.

Autophagosome–lysosome fusion is an important autophagic process for cargo degradation. ATG8 family members, tethering factors, Rab GTPases (such as RAB7), and SNARE proteins act coordinately to mediate this process. Dysfunction of autophagosome maturation is associated with various human diseases, including neurodegenerative diseases, cancer, and lysosomal storage disorders^22^. Understanding the molecular mechanisms underlying autophagosome maturation will provide new insights into the pathogenesis and treatment of these diseases. STX17 is a well-studied SNARE protein that mediates autophagosome–lysosome fusion. Upon starvation stimulus, STX17 translocates to autophagosomes, where it forms a complex with SNAP29, VAMP8, or VAMP7^23^. *STX17* inactivation prevents autophagosome–lysosome fusion. Furthermore, ATG14 and UVRAG can also promote the fusion of autophagosomes and lysosomes. ATG14 can bind to STX17, and stabilizes the STX17–SNAP29 binary *t*-SNARE complex on autophagosomes, further priming the autophagosome for VAMP8 interaction and promoting autophagosome–lysosome fusion. Our results show that RNF115 can interact with STX17 and enhance its stability, but not SNAP29 or VAMP8. We suspect that RNF115 may not bind the individual VAMP8 and SNAP29 proteins, but may associate with the STX17–SNAP29–VAMP8 complex through interacting with STX17, thereby promoting the maturation of the autophagosome or increasing the fusion rate. In addition, our results also show that RNF115 can mediate the stability of STX17. As an E3 ubiquitin ligase, RNF115 does not influence the ubiquitination of STX17, implying that another mechanism may be involved in the homeostatic regulation of STX17 protein by RNF115, which merits further investigation.

RNF115 has been reported to be overexpressed in >50% of invasive breast tumors, and is important for regulating breast cancer cell proliferation, migration, and invasion^20^. Moreover, high expression of RNF115 is associated with regional recurrence, lymph node metastasis, and unfavorable prognosis in breast cancer. Mechanistic investigations reveal that RNF115 promotes breast cancer cell proliferation through targeting the cyclin-dependent kinase inhibitor p21Waf/Cip1 for ubiquitin-dependent degradation. In the present study, it was found that the expression of RNF115 is increased in gastric adenocarcinoma samples compared to normal gastric tissue. Furthermore, the low levels of *RNF115* mRNA in gastric tissues correlated to better prognosis in patients with gastric cancer, indicating that the expression of RNF115 may be a potential independent prognostic factor for patients with gastric cancer. In vitro and in vivo investigation demonstrated that *RNF115* knockdown in gastric cancer cells can decrease cell growth and reduce tumorigenicity in a xenograft model. This phenomenon may be related to the blockade of autophagy, suggesting that the inactivation of *RNF115* exerted antitumor activity in gastric cancer cells. The relationship between autophagy and tumor growth and metastasis regulated by RNF115 requires further elucidation.

## Materials and methods

### Antibodies, plasmids, and reagents

Primary antibodies used in this study include monoclonal antibodies against RNF115 (Sigma Aldrich, St. Louis, MO, USA, 019130), SQSTM1 (MBL, Woburn, MA, USA, PM045), LC3 (Cell Signaling Technology, Danvers, MA, USA, 2775 s), ubiquitin (Cell Signaling Technology, 3936), GAPDH (Sungene, Tianjin, China, KM9002), MYC (Sungene, KM8003), and GFP (Sungene, KM8009). Secondary antibodies included DyLight 800/DyLight 680-conjugated IgG against mouse (Rockland, Philadelphia, PA, USA, 610-145-002/610-144-002) or rabbit (Rockland, 611-145-002/611-144-002).

GFP-STX17, MYC-VAMP8, and GFP-SNAP29 plasmids were kindly provided by Dr. Hong Zhang (Institute of Biophysics, Chinese Academy of Sciences). RNF115 was constructed in the pEGFP-C1 vector and mCherry-C1 vector as GFP-RNF115 or mCherry-RNF115, respectively. GFP-LC3 and mTagRFP-GFP-LC3 plasmids were produced in our lab. CQ (C6628) and MG132 (C2211) were purchased from Sigma Aldrich.

### Cells culture and transfection

HeLa cells, BGC823 cells, and HEK293T cell lines from American Type Culture Collection were cultured in Dubelcco’s modified Eagle’s medium (Invitrogen, Carlsbad, CA, USA, 12800-017) supplemented with 10% fetal bovine serum and maintained at 37 °C in a humidified incubator with 5% CO_2_. HeLa cells stably expressing GFP-LC3 were kindly provided by Dr. Li Yu (Tsinghua University).

The *shRNA* targeting the *RNF115* gene was synthesized by GenePharma (Suzhou, China) and the targeting sequence was CGTCTGAATAGAATTAATT. The negative control *shRNA* sequence consisted of an oligonucleotide sequence with no sequence homology to any known human gene. NEOFECT^TM^ DNA transfection reagent was used for transient transfection of plasmids, according to manufacturer’s instructions. For *shRNA* transfection, cells were transfected with Lipofectamine3000 according to manufacturer’s instructions (Invitrogen, L3000015).

### Western blot analysis

Treated cells were washed twice with cold phosphate-buffered saline (PBS) solution and lysed in RIPA buffer containing a cocktail of protease inhibitors. The cells were then placed on ice for 30 min, and the lysates were centrifuged at 15,000 r.p.m. for 15 min at 4 °C. The total protein concentration was measured with a Protein Quantification Kit (BCA Assay). Equal amounts of proteins were separated on sodium dodecyl sulfate–polyacrylamide gel electrophoresis (SDS–PAGE) gels and transferred onto nitrocellulose membranes. After blocking the membrane with 5% nonfat milk for 1 h, blots were visualized using an IRDye 800CW-conjugated or Alexa Fluo 680 secondary antibody, and imaged using an Odyssey infrared imaging system (LI-COR Biosciences, Lincoln, NE, USA).

### GFP-TRAP assays

HEK293T cells were transiently cotransfected with the indicated plasmids for 24 h, then treated with MG132 (20 μM) for 4–6 h. Cell lysates were immunoprecipitated by GFP-TRAP agarose beads for 4 h at 4 °C. Then the GFP-TRAP agarose beads were washed with PBS five times and boiled for 10 min in 2 × SDS–PAGE loading buffer. The samples were analyzed by western blotting.

### Immunofluorescence, fluorescence, and confocal microscopy assays

HeLa cells were cultured in confocal dishes and transfected with the indicated plasmids. Cells were then washed with PBS and fixed in 4% PFA (dissolved in PBS) for 30 min at 37 °C. The fixed cells were permeabilized with 0.2% Triton X-100 for 15 min at 37 °C and blocked with BSA for 1 h. The cells were then incubated with primary antibodies overnight at 4 °C. After washing three times with PBS-T, cells were stained with FITC/TRITC-conjugated secondary antibodies and imaged by a Leica TCSSP8 Confocal Microscope.

For the tandem mRFP-GFP-LC3 assay, HeLa cells were transfected with mRFP-GFP-LC3 or cotransfected with the indicated plasmids for 24 h. Next, cells were fixed with 4% PFA at 37 °C for 15 min, coverslipped, sealed with nail polish, and observed under a confocal fluorescent microscope. Representative cells were randomly selected for analysis, and the numbers of GFP^+^ mRFP^+^ puncta per cell were calculated.

### Cell viability and colony formation assays

BGC823 cells stably transduced with *pLVX-shcontrol* or *pLVX-shRNF115* were treated with serum deprivation for 18 h. Cells were then cultured in complete culture medium for indicated time. Cell viability assays were performed using the CellTiter 96 AQueous One Solution Cell Proliferation Assay (Promega, G1111) according to the manufacturer’s instructions. Absorbance at 490 nm was measured on an EL-311SX ELISA Reader (Bio-Tec Instruments, USA). Cell viability was calculated as follows: cell viability = absorbance of test group/absorbance of control cell group × 100%. Each experiment was performed in biological triplicate and independently repeated three times.

For the colony formation assay, cells were plated in triplicate at 100, 200, and 400 cells/well, and cultured for 2 weeks in 24-cell plates. Then cells were fixed with methanol for 15 min and stained with crystal violet for 30 min. Colonies were counted and photographed.

### Tumorigenicity in nude mice

A nude mouse xenograft model was established using 8-week-old male BALB/c nude mice (Experimental Animal Center, Peking University Health Sciences Center, Beijing, China). Mice were housed and maintained in a pathogen-free facility, and all experimental procedures were approved by the Institutional Authority for Laboratory Animal Care of Peking University (LA2019203). BGC823 cells stably transduced with *pLVX-shcontrol* or *pLVX-shRNF115* were subcutaneously injected in the right axilla of randomized BALB/c nude mice (3 × 10^6^ cells/mouse, *n* = 6). Mice were euthanized at 19 days after cell inoculation. Tumors were excised and photographed.

### Peritoneal metastasis assay in nude mice

BGC823 cells stably transduced with *pLVX-shcontrol* or *pLVX-shRNF115* (6 × 10^6^ cells/mouse, *n* = 6) were injected into the peritoneum of randomized BALB/c nude mice (8-week-old male). The body weight of the mice was monitored every 4 days after injection. At 28 days after inoculation, the mice were euthanized and metastatic nodules were observed and photographed.

### Statistics analysis

Data are presented as the mean ± s.d. Differences between groups were analyzed using the Student’s *t* test for continuous variables. Statistical significance in this study was set at *p* < 0.05. All reported *p* values are two sided.

## Supplementary information

Supplementary figure legends

FIGURE S1

FIGURE S2

FIGURE S3

FIGURE S4

FIGURE S5

FIGURE S6

FIGURE S7
