# Supplementary material for: RNF115 deletion inhibits autophagosome maturation and growth of gastric cancer
Source: Cell Death Dis. 2020 Sep 26;11(9):810. doi: 10.1038/s41419-020-03011-w (PMC7519909; doi:10.1038/s41419-020-03011-w)
Supplement: Supplementary file 1 — Supplementary figure legends [file 41419_2020_3011_MOESM1_ESM.docx]

**Supplementary data**

***RNF115* deletion inhibits autophagosome maturation and growth of gastric cancer**

Riyong Li^1,2,4^, Zhaohui Gu^1,2,4^, Xuan Zhang^1,2^, Jiahong, Yu^1,2^, Jinqiu Feng^1,2^, Yaxin Lou^3^, Ping LV^1^, Yingyu Chen^1,2,^*

^1^ Department of Immunology, School of Basic Medical Sciences, Peking University; NHC Key Laboratory of Medical Immunology( Peking University ), Beijing, China.

^2^ Center for Human Disease Genomics, Peking University, Beijing, China.

^3^Medical and Healthy Analytical Center, Peking University, Beijing, China.

*Corresponding author: Yingyu Chen, Department of Immunology, Peking University School of Basic Medical Sciences, 38 Xueyuan Road, Beijing 100191, China; E-mail: [yingyu_chen@bjmu.edu.cn](mailto:yingyu_chen@bjmu.edu.cn)

^4^ These authors contributed equally to this work.

**Materials and Methods**

**Quantitative real-time PCR (qRT-PCR)**

Total RNA was extracted from Hela cells with the TRIzol Reagent. cDNA was synthesized using the ThermoScript RT-PCR System. Real-time PCR was performed using SYBR Premix Ex Taq. The primers for q-RT-PCR were as follows: primers for RNF115, forward-5’-CGGCAGTCGGATAGACAATAC-3’and reverse-5’-TGTCAG GACGAGAACTTCCTC-3’; primers for GAPDH, forward-5’- ACAACTTTGGTATC GTGGAAGG-3’ and reverse 5’- GCCATCACGCCACAGTTTC-3’.

**Tissue Microarray and Immunohistochemistry**

A gastric cancer tissue microarray (ID: ST8014) was purchased from ChaoYing Biotechnology CO., LTD (Xian, China). The expression of RNF115 in the tissues was evaluated by immunohistochemical staining with an RNF115 specific antibody , using the DAKO Cytomation EnVision System-HRP (DAB) detection kit.

**Supplementary Figure legends**

**Fig.S1 Knockdown of *RNF115* increases the accumulation of LC3B-II and SQSTM1.** (**a**) HeLa cells were transfected with *shcontrol* or *shRNF115* for 48 h, with or without rapamycin (5 μM) for 4 h. The levels of LC3B and SQSTM1 were analyzed by western blotting. (**b** and **c**) GFP-LC3B HEK293T cells were treated with *shcontrol* or *shRNF115* for 48 h, with or without rapamycin (5 μM) for 4 h, then the levels of GFP-LC3B, endogenous LC3B and SQSTM1 were analyzed by western blotting. (**d**) BGC823 cells were transfected with *shcontrol* or *shRNF115* for 48 h, with or without rapamycin (5 μM) for 4 h, then the levels of LC3B and SQSTM1 were analyzed by western blotting. (**e**) MCF7 cells were transfected with *shcontrol* or *shRNF115* for 48 h, with or without CCCP (10 μM) for 4 h, then the levels of LC3B and SQSTM1 were analyzed by western blotting.

**Fig.S2 Overexpression of RNF115 decreases the accumulation of LC3B-II and SQSTM1.** (**a**) HeLa cells were transfected with GFP-vector or GFP-RNF115 for 24 h, with or without CQ (50μM) for 4 h. The levels of LC3B and SQSTM1 were analyzed by western blotting. (**b**) HeLa cells were transfected with *shcontrol* or *shRNF115* for 48 h, then transfected with GFP-vector or GFP-RNF115 for 24 h as indicated, the levels of SQSTM1 were analyzed by western blotting.

**Figure S3 Knockdown of *RNF115* does not affect the distribution of DFCP1.** (**a**) Hela cells were transfected with *shRNA* or *shRNF115* for 24 h, then with GFP-DFCP1 for another 24 h and EBSS treatment for 2 h before confocal microscopy analysis. (**b**) The quantification of GFP-DFCP1 puncta/cell was calculated. Data are means±SD of at least 50 cells scored. Scar bar, 25 μm. n.s, not significance.

**Figure. S4 Knockdown of *RNF115* promotes the decay of STX17 protein.** HEK293T cells were transfected with *sicontrol* or *siRNF115* for 24 h, then treated with cycloheximide (CHX, 50 μg/ml) for the indicated times. The levels of endogenous STX17 and RNF115 were analyzed by western blotting.

**Figure. S5 RNF115 had no obvious effect on STX17 polyubiquitination.** The cotransfection of HEK293T cells is shown in the figure. After 24 h, these cells were treated with MG132 for 6 h, then the cell lysates were immunoprecipitated with an anti-GFP antibody, and the Western blot was probed with an anti-HA antibody to detect ubiquitinated STX17 (left panel). Simultaneously, 10% cell lysates were used to western blotting (right panel).

**Figure. S6 High RNF115 expression results in a worse prognosis in gastric cancer patients. (a and c)** The expression level of *RNF115* and *STX17* mRNA in patients with gastric carcinoma was analyzed by the Gene Expression profiling Interactive Analysis database. (**b and d)** The levels of *RNF115* and *STX17* mRNA in gastric cancer patients were analyzed by the Kaplan-Meier Plotter database. (**e**)The correlation analysis between RNF115 and STX17 expression in gastric cancer (http://gepia.cancer-pku.cn/detail.php?gene=&clicktag=boxplot). (**f)** The expression level of RNF115 protein in non-tumor tissue adjacent and tumor tissue were analyzed by immunohistochemistry.

**Figure S7 Knockdown of *RNF115* inhibits autophagic flux in** **BGC823 gastric cancer cells. (a)** BGC823 cells were infected with lentivirus vector *pLVX-shcontrol* or *pLVX-shRNF115* for 48 h, then stable selected by puromycin (5 mg/ml) for one week. The levels of *RNF115 mRNA* were assessed by quantitative real-time PCR assay. (**b)** Cells were treated as (a), the levels of RNF115 protein were detected by western blotting. (**c)** BGC823 cells stable infected with *pLVX-shcontrol* or *pLVX-shRNF115* were cultured with or without CQ (50μM) for 4 h, then the levels of LC3B and SQSTM1 were analyzed by western blotting.
