# Supplementary figures and images for: RNF115 deletion inhibits autophagosome maturation and growth of gastric cancer
Source: Cell Death Dis. 2020 Sep 26;11(9):810. doi: 10.1038/s41419-020-03011-w (PMC7519909; doi:10.1038/s41419-020-03011-w)

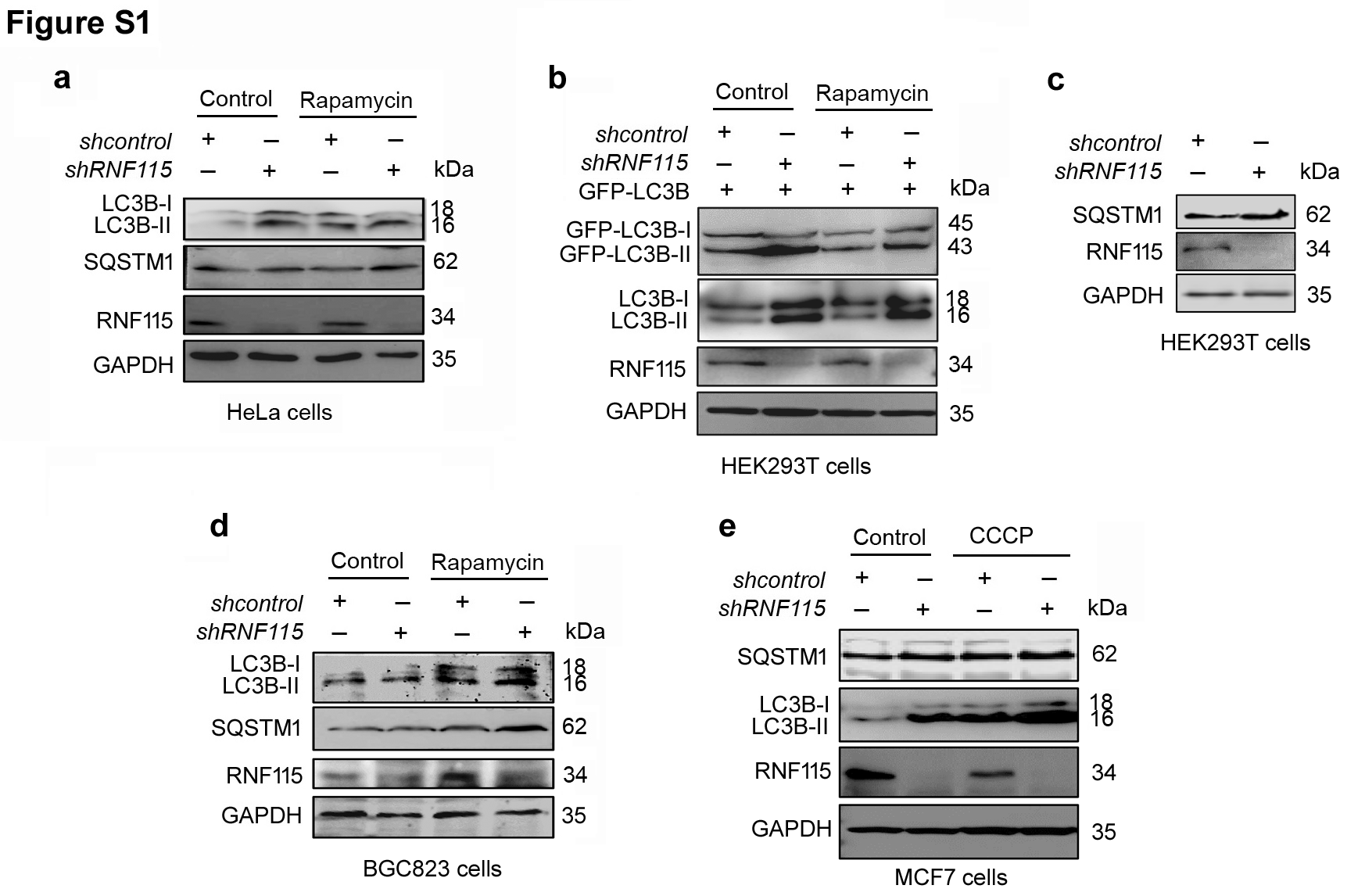

Supplement: Supplementary file 2 — FIGURE S1 [file 41419_2020_3011_MOESM2_ESM.tif]

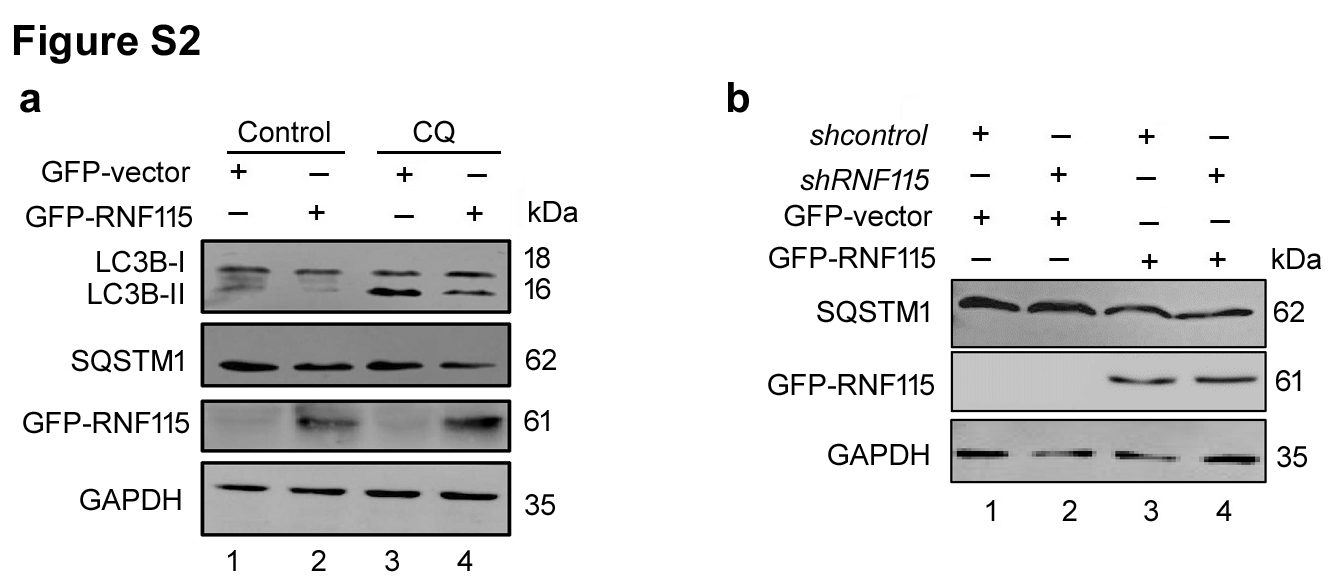

Supplement: Supplementary file 3 — FIGURE S2 [file 41419_2020_3011_MOESM3_ESM.tif]

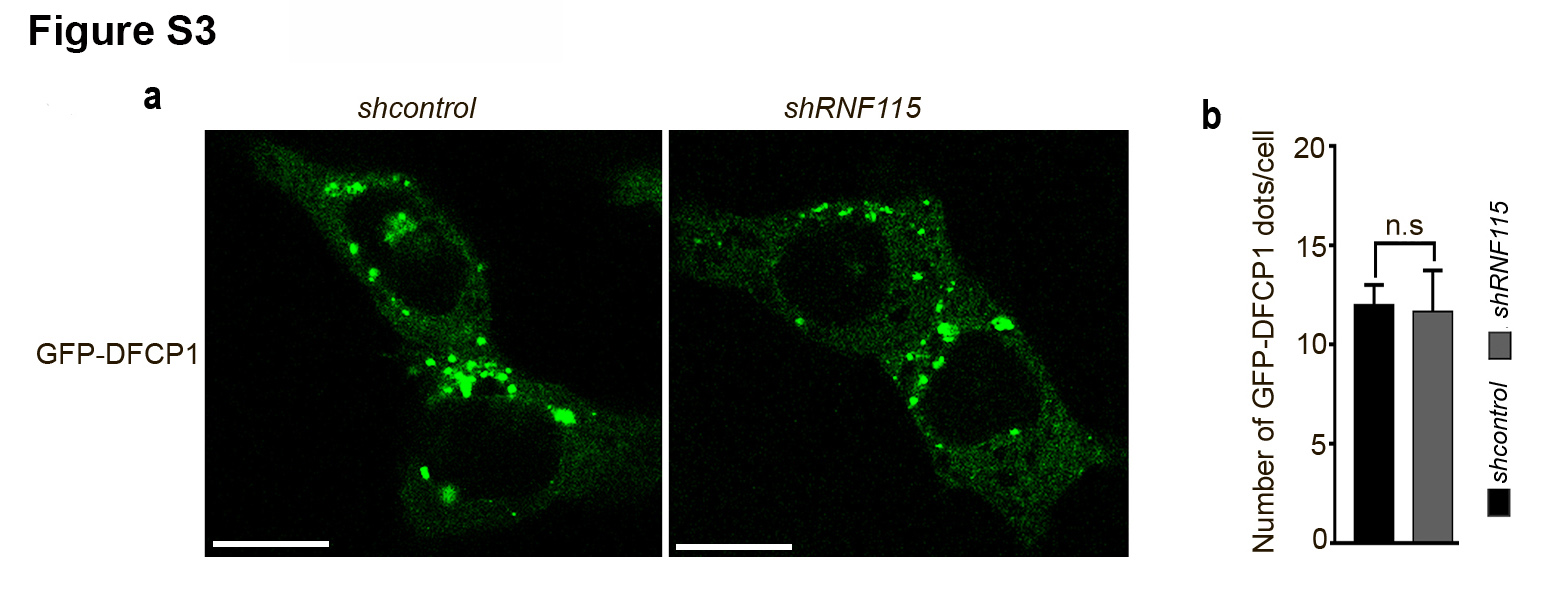

Supplement: Supplementary file 4 — FIGURE S3 [file 41419_2020_3011_MOESM4_ESM.tif]

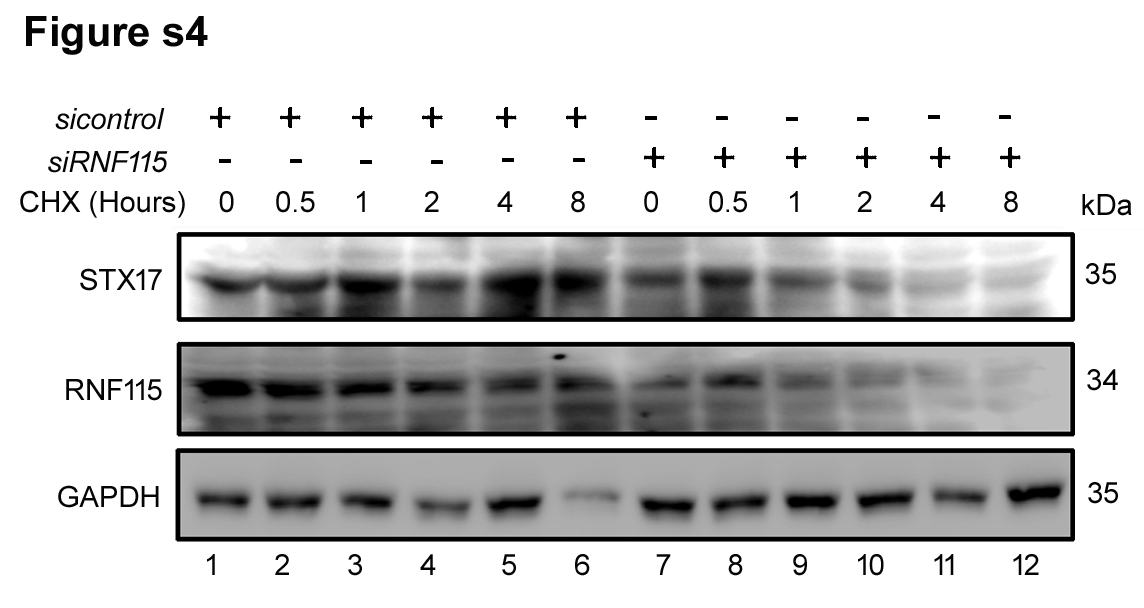

Supplement: Supplementary file 5 — FIGURE S4 [file 41419_2020_3011_MOESM5_ESM.tif]

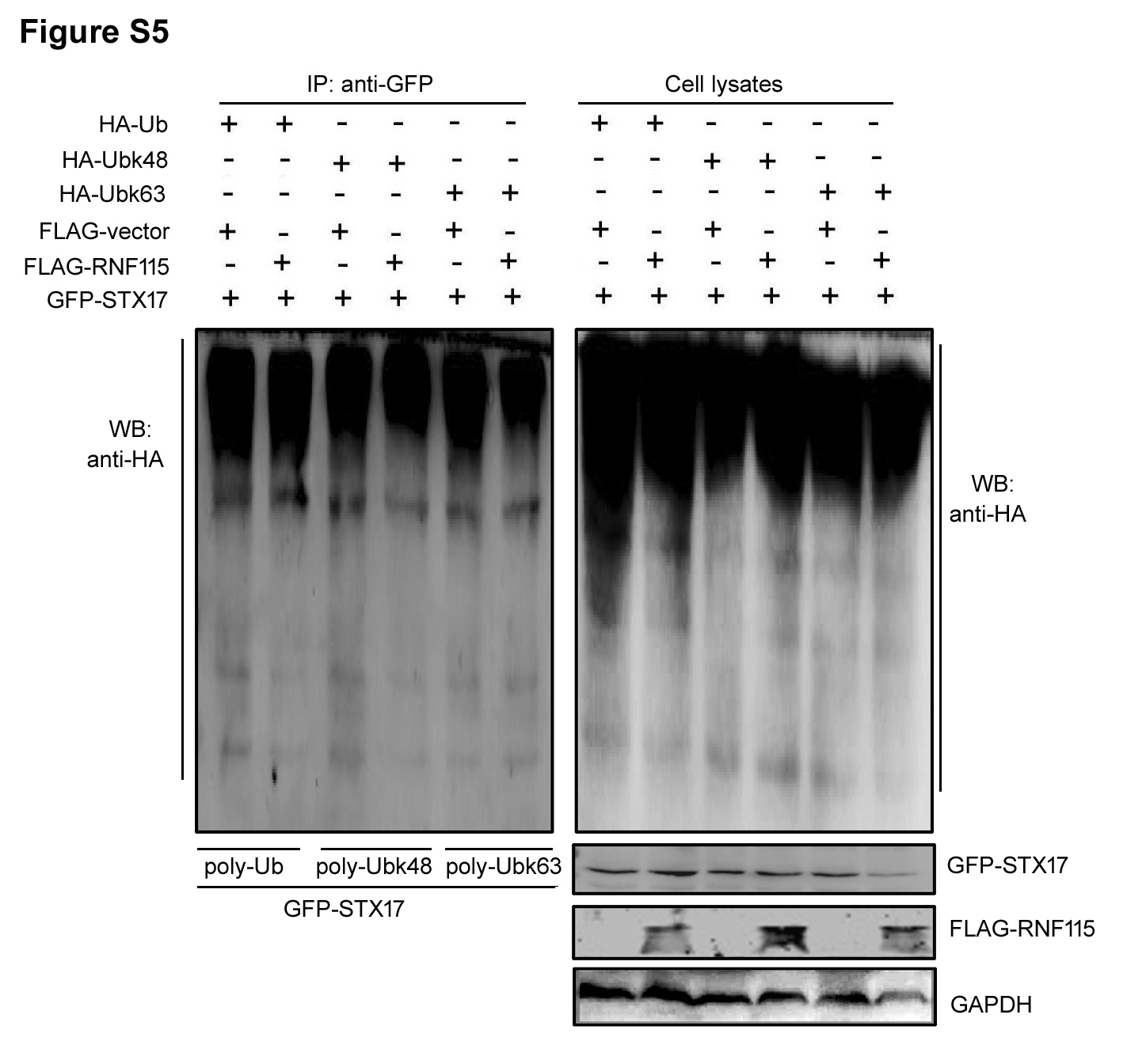

Supplement: Supplementary file 6 — FIGURE S5 [file 41419_2020_3011_MOESM6_ESM.tif]

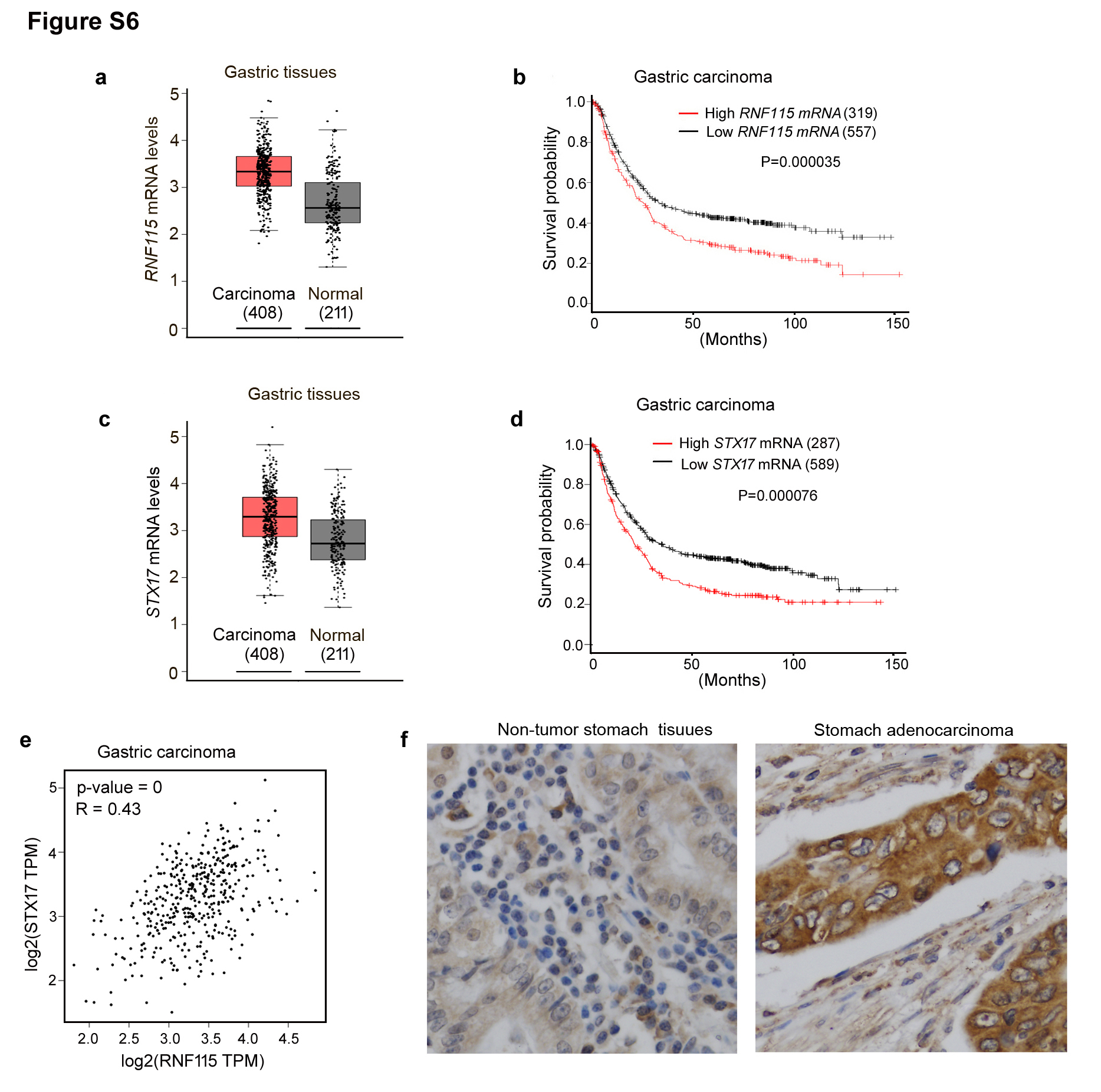

Supplement: Supplementary file 7 — FIGURE S6 [file 41419_2020_3011_MOESM7_ESM.tif]

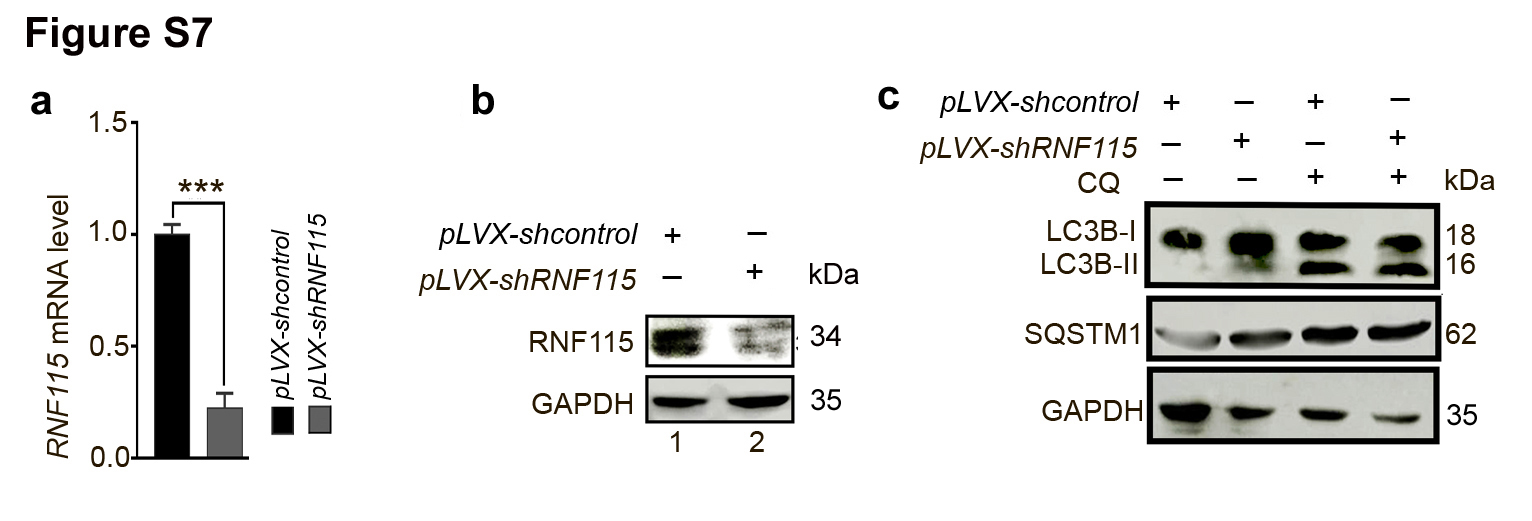

Supplement: Supplementary file 8 — FIGURE S7 [file 41419_2020_3011_MOESM8_ESM.tif]
